# Predictive value of neutrophil to lymphocyte ratio for clinical outcome in patients with atrial fibrillation: a systematic review and meta-analysis

**DOI:** 10.3389/fcvm.2024.1461923

**Published:** 2024-09-26

**Authors:** Lei Peng, Li Liu, Miaomiao Chai, Zhonggui Cai, Deqi Wang

**Affiliations:** ^1^ Department of Cardiology, Linping Hospital of Integrated Traditional Chinese and Western Medicine, Hangzhou, China; ^2^ Department of Cardiology, Jinan Integrated Traditional Chinese and Western Medicine Hospital, Jinan, China; ^3^ Department of Interventional Cardiology, Shandong Healthcare Group Zaozhuang Hospital, Zaozhuang, China; ^4^ Department of Interventional Cardiology, Zaozhuang Municipal Hospital, Zaozhuang, China

**Keywords:** neutrophil-to-lymphocyte ratio, atrial fibrillation, prognosis, systematic review, meta-analysis

## Abstract

**Background:**

The association between the Neutrophil-to-Lymphocyte Ratio (NLR) and the prognosis of Atrial Fibrillation (AF) has been extensively studied, yet clinical outcomes have varied. Consequently, this analysis was undertaken to explore the link between NLR and the prognostic markers of AF.

**Methods:**

We conducted an exhaustive search across electronic databases, including PubMed, Embase, Web of Science, and the Cochrane Library, to investigate the correlation between the NLR and indicators of adverse clinical outcomes associated with AF from the database establishment date through March 31, 2024. In this study, the recurrence rate of AF was the primary outcome measure, while the secondary outcome measures were mortality, stroke, and left atrial thrombus. Odds ratio (OR), relative risk (RR), hazard ratio (HR) and standard mean difference (SMD) with a 95% confidence interval (CI) were integrated for assessment, and the stability of prognostic outcomes and publication bias were verified by sensitivity analysis and Egger's test, respectively. Subgroup analyses were performed to pinpoint the sources of heterogeneity.

**Results:**

This analysis included 20 studies, encompassing a total of 59,256 patients. Our statistical analysis of both categorical and continuous variables revealed that an elevated NLR was significantly associated with increased risks in AF patients for recurrence (categorical variable: OR = 1.39, 95% CI = 1.21–1.60; continuous variable: SMD = 0.49, 95% CI = 0.24–0.74), mortality (categorical variable: OR = 1.87, 95% CI = 1.59–2.20), stroke (categorical variable: OR = 1.56, 95% CI = 1.13–2.17; continuous variable: SMD = 0.77, 95% CI = 0.63–0.91), and left atrial thrombus (categorical variable: OR = 1.87, 95% CI = 1.27–2.75; continuous variable: SMD = 0.59, 95% CI = 0.30–0.89). Subgroup analyses found that high NLR was significantly linked to AF recurrence when the NLR was >3. High NLR was significantly linked to the risk of stroke in AF when the NLR was ≤3.

**Conclusions:**

This study suggested that a high NLR is significantly linked to prognostic risk markers of AF, and NLR may be an effective biomarker for the prognosis of AF in clinical practice.

**Systematic Review Registration:**

PROSPERO (CRD42024530970).

## 1 Introduction

Atrial fibrillation (AF) is one of the most common arrhythmias encountered in clinical practice. It may significantly increase the risk of stroke, heart failure, cardiac arrest, mortality and other adverse outcomes (1). Its incidence is on the rise annually, leading to a considerable deterioration in patients’ quality of life. Data from the Framingham Heart Study indicates that the prevalence of AF has increased twofold over the last five decades (1, 2). The estimated count of AF cases in the United States is anticipated to grow from 1.2 million to 2.6 million between 2010 and 2030 (3). Similarly, in Europe, the number of cases could hit 14 million by the year 2060 (4). At present, AF can be treated by drugs, radiofrequency ablation and surgical intervention, but its recurrence rate is still high (5, 6). For example, La Fazia et al. found in a clinical study that HIV+ AF patients had a persistently high mid- to long-term recurrence rate after undergoing catheter ablation (CA) (7). Although there are different treatments for AF currently, there remains a lack of upstream biomarkers to predict AF prognosis effectively. Therefore, it is still a challenge to pursue effective biomarkers that can predict the clinical outcomes of AF.

A variety of inflammatory indicators, such as interleukins (IL) and C-reactive protein (CRP) have been linked to the occurrence and outcomes of AF. Among them, the neutrophil-lymphocyte ratio (NLR), derived from the comparison of neutrophil to lymphocyte counts, has gained recognition as a robust predictor for the prognosis of AF (8). White blood cell (WBC) counts, along with their subtypes, serve as measures of inflammatory activity. Within the leukocyte spectrum, an elevated neutrophil level suggests a nonspecific inflammatory response, while lymphopenia is indicative of physiological stress and a diminished health condition. Consequently, the NLR offers insight into the equilibrium between these two cell types, shedding light on the body's stress and inflammatory profiles (9). Evidence showed that NLR has good predictive value for progression and clinical outcome of cardiovascular disorder (10). For instance, a significant correlation was identified between elevated preoperative NLR and the recurrence of AF following Cox-Maze IV procedure (CMP-IV) (11). Another large, randomized study reported that increased NLR is associated with a heightened risk of cardiovascular-related events and death in individuals with AF (12). Meanwhile, some studies have debated whether NLR could predict AF prognosis. For example, a clinical study reported that NLR levels did not have predictive value for AF recurrence based on the comparison of NLR levels before and after cryoballoon ablation (13). For this, this comprehensive and precise meta-analysis was conducted to identify the precise predictive function of NLR for AF.

Although recent meta-analyses of NLR on prognosis in AF have been published (14), they focused on a limited range of clinical outcomes. Therefore, this meta-analysis was performed to comprehensively integrate common outcome measures in AF, aiming to evaluate the predictive performance of NLR.

## 2 Materials and methods

### 2.1 Protocol and registration

The meta-analysis was duly registered in the Preferred Reporting Guidelines, International Prospective Register of Systematic Reviews (PROSPERO) (ID: CRD42024530970). There were no protocol deviations from the current study. Furthermore, the research was executed in full compliance with the PRISMA2020 (Preferred Reporting Items for Systematic Reviews and Meta-Analyses) guidelines (15).

### 2.2 Study selection

The study selection was guided by the following inclusion criteria: (1) subjects were patients with AF, including various types such as paroxysmal atrial fibrillation, persistent atrial fibrillation, long-standing persistent atrial fibrillation, and permanent atrial fibrillation; (2) the study encompassed a variety of research designs, including randomized controlled trials, cohort studies, and case-control studies.; (3) human subjects were involved; (4) test data included NLR; (5) reported AF outcome measures include recurrence rate, mortality, stroke, and left atrial thrombus, with OR, RR, or HR and 95% CI, as well as SMD, or information that allows for these calculations.”; (6) the study published in English. Exclusion criteria included: (1) case reports, letters, conference abstracts, reviews, and comments; (2) animal experiments; (3) studies with duplicated or overlapping data; and (4) non-English language articles.

### 2.3 Literature retrieval

An extensive search of online databases from PubMed, Embase, Web of Science, and the Cochrane Library was conducted from database establishment date through March 31, 2024. The keywords were neutrophil and Lymphocyte and ratio and atrial fibrillation. PubMed retrieval formula is as follows: ((((“Neutrophils"[Mesh]) OR ((((((((((((((Neutrophil) OR (Leukocytes, Polymorphonuclear)) OR (Leukocyte, Polymorphonuclear)) OR (Polymorphonuclear Leukocyte)) OR (Polymorphonuclear Leukocytes)) OR (Polymorphonuclear Neutrophils)) OR (Neutrophil, Polymorphonuclear)) OR (Polymorphonuclear Neutrophil)) OR (LE Cells)) OR (Cell, LE)) OR (LE Cell)) OR (Neutrophil Band Cells)) OR (Band Cell, Neutrophil)) OR (Neutrophil Band Cell))) AND ((“Lymphocytes"[Mesh]) OR (((((Lymphocyte) OR (Lymphoid Cells)) OR (Cell, Lymphoid)) OR (Cells, Lymphoid)) OR (Lymphoid Cell)))) AND (ratio)) AND ((“Atrial Fibrillation"[Mesh]) OR (((((((((((((((((((((((((Atrial Fibrillations) OR (Fibrillation, Atrial)) OR (Fibrillations, Atrial)) OR (Auricular Fibrillation)) OR (Auricular Fibrillations)) OR (Fibrillation, Auricular)) OR (Fibrillations, Auricular)) OR (Persistent Atrial Fibrillation)) OR (Atrial Fibrillation, Persistent)) OR (Atrial Fibrillations, Persistent)) OR (Fibrillation, Persistent Atrial)) OR (Fibrillations, Persistent Atrial)) OR (Persistent Atrial Fibrillations)) OR (Familial Atrial Fibrillation)) OR (Atrial Fibrillation, Familial)) OR (Atrial Fibrillations, Familial)) OR (Familial Atrial Fibrillations)) OR (Fibrillation, Familial Atrial)) OR (Fibrillations, Familial Atrial)) OR (Paroxysmal Atrial Fibrillation)) OR (Atrial Fibrillation, Paroxysmal)) OR (Atrial Fibrillations, Paroxysmal)) OR (Fibrillation, Paroxysmal Atrial)) OR (Fibrillations, Paroxysmal Atrial)) OR (Paroxysmal Atrial Fibrillations))). The complete retrieval formulas of the other databases are provided in Supplementary Table S1.

### 2.4 Data extraction

Two investigators (LP and DW) independently evaluated eligible studies and performed data extraction from those included studies. Any discrepancies were addressed through discussions with all contributing co-authors. The data extracted encompassed details such as first author name, publication year, study period, country/region, test timing, sample size, and subjects’ age, sex, body mass index (BMI), blood pressure, left ventricular ejection fraction (LVEF), and left atrial area, follow-up time, cut off, and OR or RR or HR, and 95% CI and SMD. The primary endpoint of interest was the recurrence of AF, while secondary endpoints included mortality, incidence of stroke, and the presence of left atrial thrombus.

### 2.5 Quality assessment of the studies included

The included study quality was appraised using the Newcastle-Ottawa Scale (NOS), with two independent evaluators (LP and LL) conducting the assessment (16) across three domains: selection (4 points), outcome and adequacy of follow-up (3 points), and comparability (2 points). The NOS scoring system allocates a total of 9 points, with studies earning 7 points or more classified as high quality.

### 2.6 Statistical analysis

Pooled effect values and 95% CI were determined by a random-effects model to estimate the prognostic effect of NLR in patients with AF. Categorical variables were pooled by OR and continuous variables were pooled by SMD. The heterogeneity between studies was evaluated by Higgins *I^2^* and Cochran's Q test. I^2^ > 50% and *P* < 0.1 meant significant heterogeneity. Sensitivity analyses and subgroup analyses stratified by factors were used to confirm the robustness of prognostic outcome measures in AF and to explore potential sources of heterogeneity. We utilized funnel plots and conducted Egger's test to assess publication bias. All data were statistically analyzed using STATA 15.0 and Review Manager 5.4. A *P*-value < 0.05 was set as the threshold for statistical significance.

## 3 Results

### 3.1 Characteristics of the included studies

**Figure 1:**
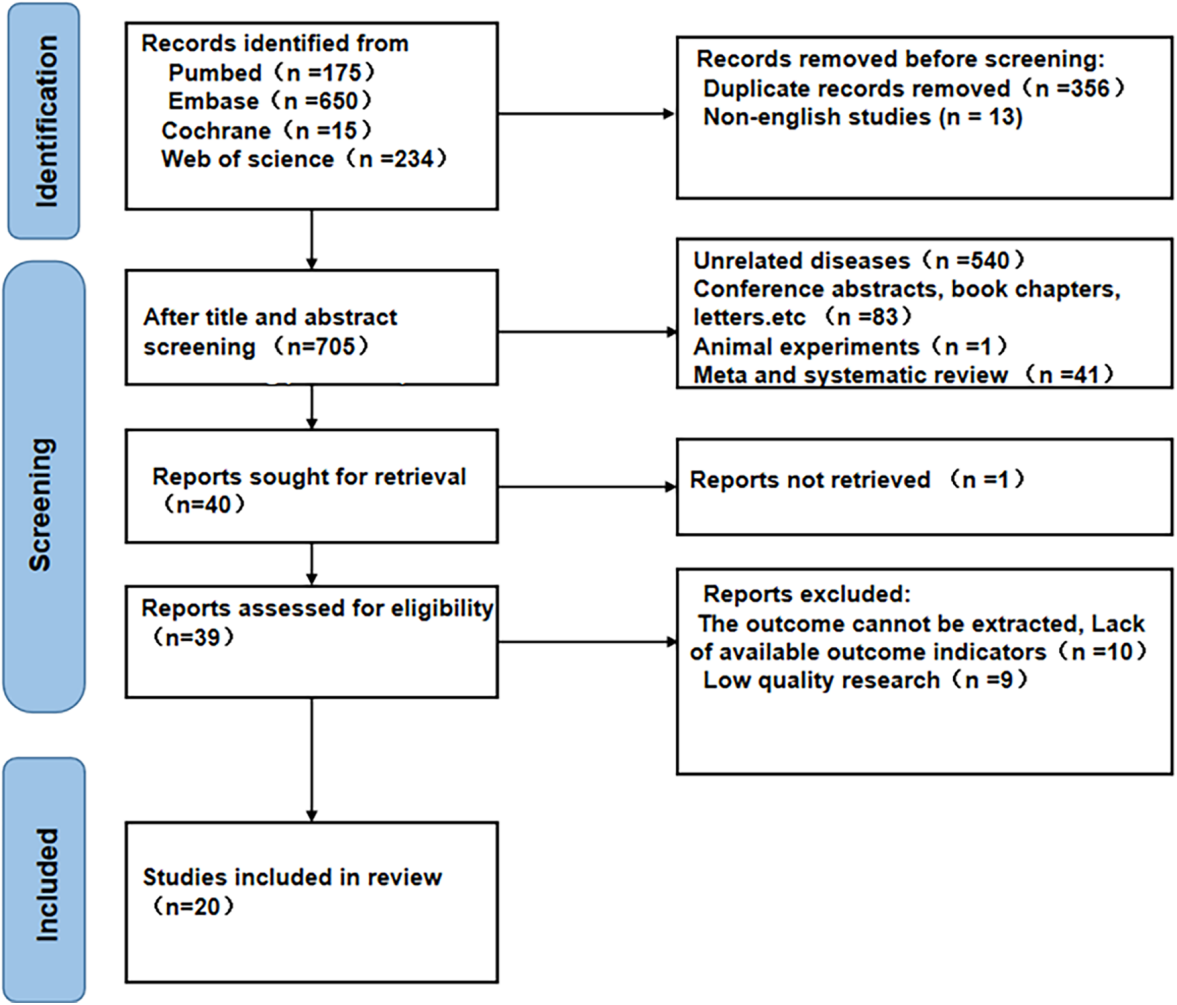
Literature screening flow chart.

Initially, the search across the four databases yielded a total of 1,074 articles. Among them, 356 duplicated and 13 non-English articles were excluded, and after reading the titles and abstracts of the others, 665 studies were further excluded. The remaining 40 studies were then read in full. Of these, 20 studies were removed: nine due to low quality, one due to the absence of a retrieval report, four because NLR data could not be extracted, and six because the outcome measures were not specified. Ultimately, 20 studies (8, 12, 17–34) involving 59,256 subjects were included in this meta-analysis (Figure 1).

Among the 20 studies, nine originated from research conducted in China (20–22, 26, 27, 29, 30, 32, 34), six in Turkey (8, 17, 19, 23, 25, 33), one each in Greece (18), South Korea (24), the United States (12), Israel (28), and Japan (31). Three (18, 21, 24) of these studies had two cohorts, and therefore a total of 23 studies were included. Twenty-two studies were retrospective (8, 17–34), with the remaining one (12) being prospective. All studies were published within the timeframe from 2013 to 2024. Eighteen of these studies provided data on the NLR and dichotomous variable data on prognostic outcome measures in AF (8, 12, 17–25, 27–31, 33, 34), with one study (12) containing both dichotomous data on mortality and stroke and another one (21) containing two sets of dichotomous data before and after treatment. Eighteen studies (8, 17–22, 24–27, 30, 32–34) reported NLR and continuous variable data on prognostic outcome measures in AF, three (18, 21, 24) of which included two pre- and post-treatment cohort studies. Regarding the measurement of NLR, 16 studies (8, 17, 19, 20, 22, 23, 25–34) investigated NLR at baseline, one (12) investigated NLR after treatment, and three (18, 21, 24) investigated NLR both at baseline and after treatment, and the baseline characteristics are fully described in Table 1.

**Table 1:** Basic characteristics of the included studies.

Author	Years	Study period	Region	Study design	Population	Time of test	Follow-up	No.of patients	Gende		Mean/median Age	BMI	LVEF (%)	SBP (mmHg)	DBP (mmHg)	LAD (mm)	cut off
									Male	Female							
Kus et al. (17)	2022	2015–2020	Turkey	Cohort studies	Permanent AF	Before treatment	1 year	99	54	45	56.7 ± 11.3	NA	60	NA	NA	41.5 ± 4.6	NA
Im et al.(a) (24)	2013	NA	Korea	Cohort studies	Patients with paroxysmal or persistent AF undergoing PTCA	Before treatment	25.2 ± 14.5 months	499	367	132	56.3 ± 11.3	24.8 ± 2.78	62.8 ± 8.83	NA	NA	41.8 ± 6.82	NA
Im et al.(b) (24)	2013	NA	Korea	Cohort studies	Patients with paroxysmal or persistent AF undergoing PTCA	After treatment	25.2 ± 14.5 months	499	367	132	56.3 ± 11.3	24.8 ± 2.78	62.8 ± 8.83	NA	NA	41.8 ± 6.82	5.6
Bazoukis et al.(a) (18)	2019	2014–2017	Greece	Cohort studies	Consecutive patients undergoing AF catheter ablation	Before treatment	26.2 ± 12.1 months	346	224	122	59 ± 11	NA	NA	NA	NA	NA	NA
Bazoukis et al.(b) (18)	2019	2014–2017	Greece	Cohort studies	Consecutive patients undergoing AF catheter ablation	After treatment	26.2 ± 12.1 months	346	224	122	59 ± 11	NA	NA	NA	NA	NA	3.9
Canpolat et al. (19)	2013	2010–2012	Turkey	Cohort studies	AF patients undergoing cryoballoon technique	Before treatment	19.0 ± 6.6 months	251	131	120	54.12 ± 10.9	25.5 ± 5.8	64.5 ± 6.0	NA	NA	38.5 ± 5.36	3.15
Ding et al. (20)	2021	2017–2019	China	Cohort studies	Patients with paroxysmal or persistent NVAF	Before treatment	1 year	263	153	110	62 (53–69)	25.28 ± 3.15	64.46 ± 6.40	NA	NA	42.43 ± 5.99	2.33
Guo et al.(a) (21)	2014	NA	China	Cohort studies	Lone AF patients undergoing catheter ablation	Before treatment	30.5 ± 5.3 months	379	278	101	49.7 ± 6.6	24.06 ± 4.39	63.19 ± 7.59	NA	NA	38.09 ± 5.71	NA
Guo et al.(b) (21)	2014	NA	China	Cohort studies	Lone AF patients undergoing catheter ablation	After treatment	30.5 ± 5.3 months	379	278	101	49.7 ± 6.6	24.06 ± 4.39	63.19 ± 7.59	NA	NA	38.09 ± 5.71	5.15
Karavelioğlu et al. (8)	2015	2006–2013	Turkey	Cohort studies	AF patients who recovered sinus rhythm after amiodarone treatment	Before treatment	21.6 ± 13.9 months	218	92	126	64.1 ± 14.6	NA	58.54 ± 11.5	118.4 ± 12.8	72.3 ± 9.80	38.97 ± 7.27	NA
Luo et al. (22)	2022	2015–2018	China	Cohort studies	Patients with persistent AF undergoing surgery	Before treatment	7 days	120	43	77	59.64 ± 58.03	NA	55.66 ± 4.53	NA	NA	52.08 ± 8.88	5.91
Aribas et al. (23)	2013	2009–2011	Turkey	Cohort studies	persistent AF patients	Before treatment	6 months	149	69	80	59.6 ± 10	28.23 ± 4.0	54.46 ± 9.58	123.31 ± 20.59	76.15 ± 11.63	43.46 ± 4.84	2.38
Ertas et al. (24)	2013	NA	Turkey	Case control studies	NVAF patients with or without thromboembolic stroke	Before treatment	NA	126	52	74	70 ± 10.2	NA	54.23 ± 10.55	132.57 ± 18.63	78.54 ± 10.91	46.69 ± 5.99	3.17
Guo et al. (26)	2024	2022–2023	China	Case control studies	NVAF patients	Before treatment	NA	301	146	155	77.03 ± 8.93	NA	62.35 ± 6.43	136.84 ± 22.57	81.11 ± 14.30	43.84 ± 5.10	NA
Shi et al. (27)	2023	NA	China	Case control studies	NVAF patients with or without cardiogenic cerebral embolism	Before treatment	NA	925	486	439	72	NA	NA	NA	NA	NA	NA
Saliba et al. (28)	2015	2012.1–2012.12	Israel	Cohort studies	All adult AF patients	Before treatment	12 months	32,912	15,932	16,980	73.2 ± 13.6	NA	NA	NA	NA	NA	3
Jr et al. (12)	2023	NA	USA	Cohort studies	AF patients treated with oral anticoagulants	After treatment	2.8 years	19,697	12,131	7,566	65	NA	NA	NA	NA	NA	4
Wu et al. (29)	2021	2010–2015	China	Cohort studies	AF patients	Before treatment	3.32 years	1,269	655	614	63.5	24.1 ± 3.7	56.5 ± 11.6	122.9 ± 20.0	74.2 ± 12.3	44.3 ± 10.2	3.59
Deng et al. (30)	2023	2019–2021	China	Case control studies	Patients diagnosed with NVAF by TEE were enrolled	Before treatment	NA	569	370	199	62.09 ± 11.52	NA	62.91 ± 8.79	NA	NA	40.19 ± 5.80	2.57
Fukuda et al. (31)	2018	2014–2016	Japan	Case control studies	Paroxysmal NVAF patients	Before treatment	NA	183	127	56	64 ± 9	NA	61 ± 6	139 ± 17	74 ± 12	38 ± 6	2.5
Tang et al. (32)	2022	2016–2020	China	Case control studies	NVAF patients	Before treatment	NA	207	115	92	65.81 ± 7.02	24.60 ± 1.35	67.20 ± 4.24	NA	NA	38.58 ± 3.32	1.85
Yalcin et al. (33)	2015	2009–2012	Turkey	Case control studies	NVAF patients	Before treatment	NA	309	145	164	70.1 ± 9.8	NA	NA	NA	NA	NA	2.59
Zhou et al. (34)	2023	NA	China	Case control studies	Patients with valvular AF	Before treatment	NA	434	157	277	56.94 ± 9.12	NA	56.92 ± 7.89	NA	NA	55.01 ± 11.35	2.66

AF, atrial fibrillation; LVEF, left ventricular ejection fraction; LAD, left atrium diameter; SBP, systolic blood pressure; DBP, diastolic blood pressure; NVAF, nonvalvular atrial fibrillation; TEE, transesophageal echocardiography.

### 3.2 Study quality of the studies included

The NOS scores for the included studies ranged between 7 and 8 points, signifying a high level of study quality. Detailed NOS scores are presented in Supplementary Tables S2, S3.

### 3.3 Analysis results of meta-analysis

#### 3.3.1 NLR and recurrence rate

**Figure 2:**
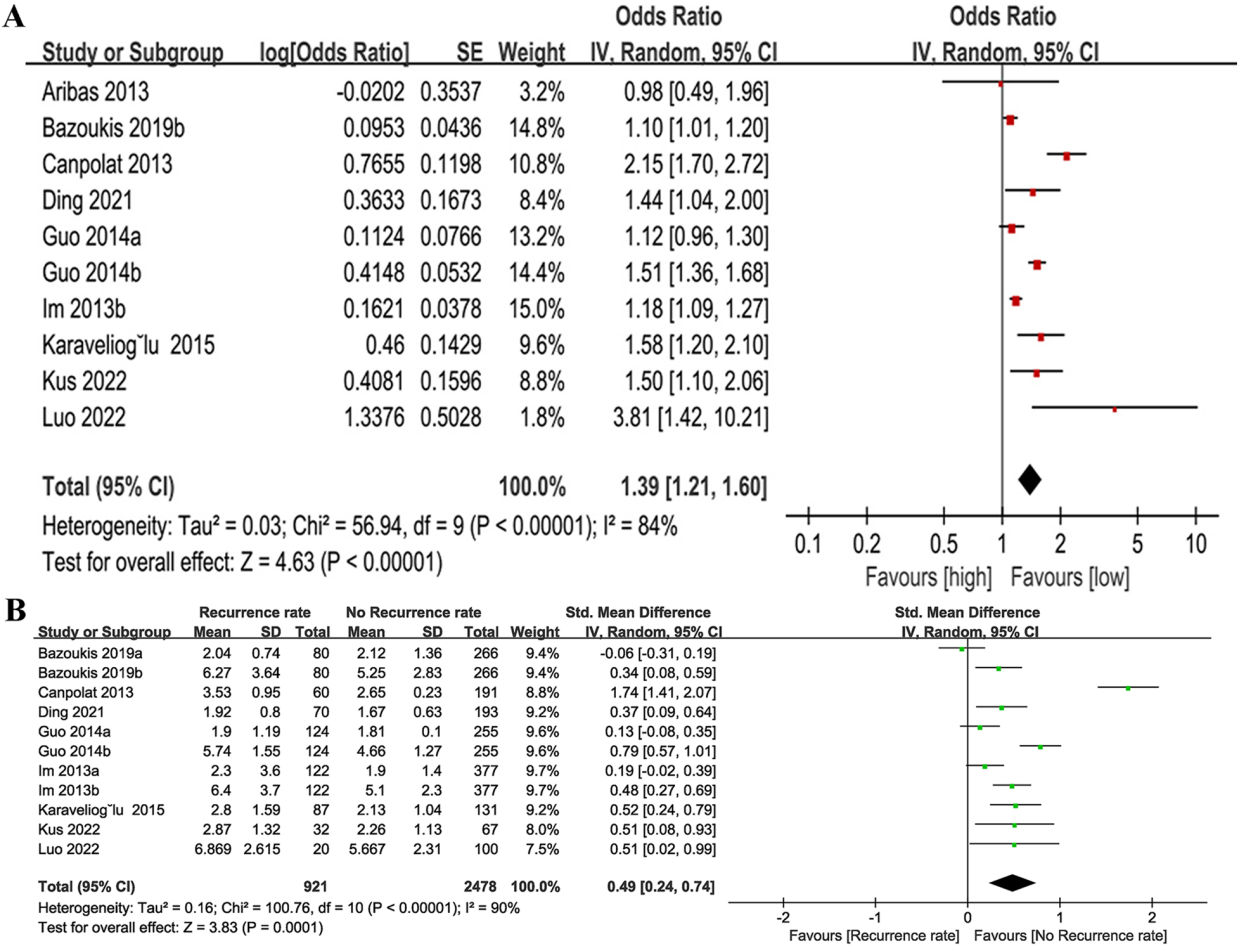
Forest plot of the association between NLR and recurrence rate as categorical variable **(A)** and continuous variable **(B).**

The relationship between NLR and recurrence rate (categorical variable) was investigated. Ten cohort studies were incorporated into the analysis in total, which exhibited significant heterogeneity (I^2^ = 84%, *P* < 0.00001) (Figure 2A). Of these studies, seven provided only baseline NLR values and three provided only post-treatment NLR values, the combined result was OR = 1.39, 95% CI = 1.21–1.60, *P* < 0.00001, indicating a higher NLR was significantly associated with higher AF recurrence rate (Figure 2A). Subgroup analyses were performed before and after treatment, follow-up time, country/region, age and NLR cut off. The findings indicated that there were also notable differences in NLR and AF recurrence rates before and after treatment, follow-up time, country/region, and age (*P* < 0.05 for all) (Table 2). In the treatment method subgroups, NLR remained a significant predictor of outcomes in the ablation subgroup (*P* < 0.05), but it was not a significant predictor in the cardioversion subgroup (OR = 1.36, 95% CI = 0.96–1.94, *P* = 0.08). Additionally, no significant heterogeneity was observed (I^2^ = 18%, *P* = 0.27). Taking 3 as the cut off of NLR, NLR > 3 was associated with AF prognosis (OR = 1.45, 95% CI = 1.18–1.78, *P* = 0.0004) and significant heterogeneity (I^2^ = 92%, *P* < 0.00001). NLR ≤ 3 was not significantly linked to the AF recurrence (OR = 1.34, 95% CI: 1.0–1.8; *P* = 0.05), without heterogeneity (I^2^ = 0%, *p* = 0.33). Additionally, we found that when the follow-up time was ≤12 months, age was >60 years, and the NLR cut off was ≤3, the heterogeneity in the cardioversion subgroup was all <50%, suggesting that factors such as follow-up time, age, and cut off may be the source of heterogeneity. The detailed data for the subgroup analysis are presented in Table 2.

**Table 2:** Subgroup analysis of NLR and recurrence rate and stroke in patients with AF.

Subgroup	Recurrence rate				Stroke			
	Study	OR [95%CI]	*P*-value	I^2^	Study	OR [95%CI]	*P*-value	I^2^
Total	10	1.44 [1.21–1.72]	0.0001	85%	5	1.56 [1.13–2.17]	0.007	83%
Study design								
Cohort	10				2	1.21 [0.92–1.59]	0.18	77%
Case-control	0				3	1.99 [1.51–2.61]	0.00001	0%
Follow-up								
>12 months	6	1.36 [1.16–1.59]	0.0001	90%	1	1.44 [1.1–1.88]	0.008	NA
≤12 months	4	1.50 [1.11–2.03]	0.009	39%	1	1.08[1.05–1.11]	0.00001	NA
Region								
Asia	9	1.45 [1.24–1.70]	0.00001	82%	4	1.66[1.05–2.64]	0.03	85%
Europe	1	1.1 [1.01–1.2]	0.03	NA				
America					1	1.44 [1.1–1.88]	0.008	NA
Time of test								
Before treatment	7	1.53 [1.19–1.98]	0.001	78%	4	1.66[1.05–2.64]	0.03	85%
After treatment	3	1.25 [1.05–1.48]	0.01	91%	1	1.44 [1.1–1.88]	0.008	NA
Mean/median age								
>60 years	2	1.52 [1.23–1.88]	0.0001	0%	5			
≤60 years	8	1.37 [1.17–1.60]	0.0001	87%	0			
NLR cut-off								
>3	5	1.45[1.18–1.78]	0.0004	92%	3	1.30[0.94–1.80]	0.11	74%
≤3	2	1.34[1.0–1.8]	0.05	0%	1	1.86[1.20–2.88]	0.006	NA
Treatment methods								
EC	2	1.36[0.96–1.94]	0.08	18%				
PC	1	1.58[1.20–2.10]	0.001	NA				
CA	7	1.38[1.17–1.62]	0.0001	89%				

OR, odds ratio; 95%CI, 95% confidence interval; EC, electric cardioversion; PC, pharmacological cardioversion; CA, catheter ablation.

For recurrence rate (continuous variable) analysis (Figure 2B), 11 cohort studies involving 2,175 patients were included. Eight of these studies provided pre-treatment baseline NLR and three studies provided pre- and post-treatment NLR. A considerable degree of heterogeneity was observed in the studies included (I^2^ = 90%, *P* < 0.00001). The findings indicated that patients with recurrent AF had significantly elevated NLR values compared to those without recurrence (SMD = 0.49, 95% CI = 0.24–0.74, *P* = 0.0001).

#### 3.3.2 NLR and mortality

**Figure 3:**
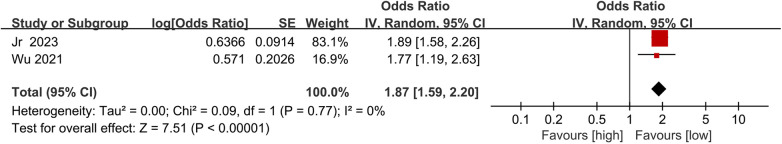
Forest plot of the association between NLR and mortality (categorical variable).

Two studies were incorporated to investigate the link between NLR and mortality: one was a prospective post-treatment study and the other was a retrospective pre-treatment study. No significant heterogeneity was observed between these two studies (I^2^ = 0%, *p* = 0.77). As shown in Figure 3, higher NLR was found to be significantly correlated with increased mortality in patients with AF (OR = 1.87, 95% CI = 1.59–2.20, *P* = 0.00001).

#### 3.3.3 NLR and stroke

**Figure 4:**
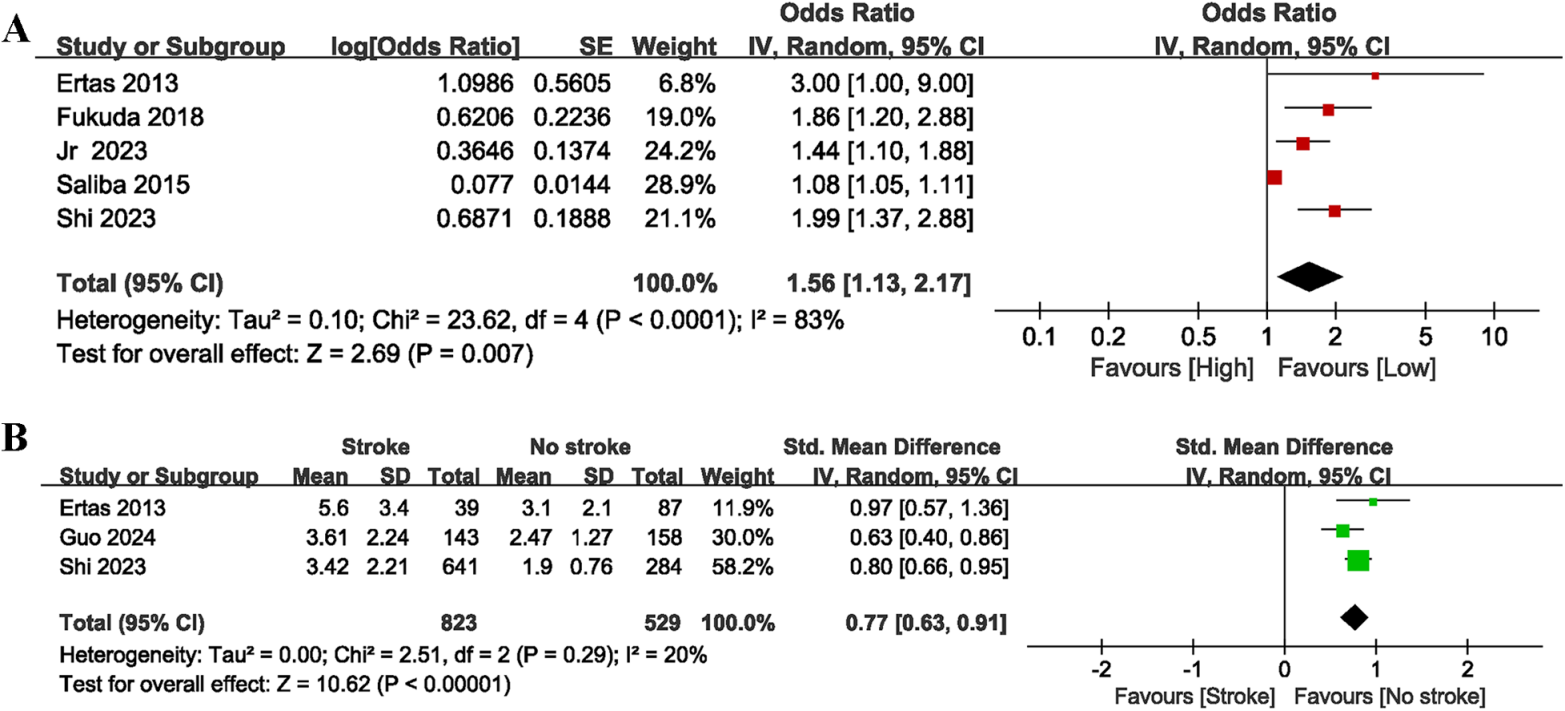
Forest plot of the association between NLR and stroke as categorical variable **(A)** and continuous variable **(B).**

In total, five studies that explored the link between NLR and the risk of stroke (categorical variable) were included, of which four provided pre-treatment baseline NLR values while one provided only post-treatment NLR values, with significant heterogeneity observed (I^2^ = 83%, *P* < 0.0001). As shown in Figure 4A, high NLR was significantly related to stroke (OR = 1.56, 95% CI = 1.13–2.17, *P* = 0.007), showing that an elevated NLR is notably correlated with an increased likelihood of stroke in individuals with AF. Subgroup analyses showed that NLR was a significant predictor for the occurrence of stroke in AF patients both before and after treatment, as well as across different follow-up times and nation/region subgroups (*P* < 0.05 for all) (Table 2). Similarly, taking 3 as the cut off of NLR, we found that in the NLR ≤ 3 subgroup, NLR was significantly associated with the risk of stroke in AF patients (OR = 1.86, 95% CI: 1.20–2.88, *P* = 0.006). In contrast, in the NLR > 3 subgroup, a significant association was not observed (OR = 1.30, 95% CI = 0.94–1.80, *P* = 0.11), and significant heterogeneity was observed (I^2^ = 74%, *P* = 0.02). In addition, the subgroup analyses found that there was no significant correlation between high NLR and stroke in AF patients in the cohort studies (OR = 1.21, 95% CI: 0.92–1.59; *P* = 0.18), with significant heterogeneity observed (I^2^ = 77%, *P* = 0.04). In contrast, controlled studies demonstrated a considerable link between high NLR and stroke in AF (OR = 1.99, 95% CI = 1.51–2.61, *P* = 0.00001), with no significant heterogeneity observed (I^2^ = 0%, *P* = 0.73), which indicated that study type may be one of the sources of heterogeneity.

For stroke (continuous variable) analysis (Figure 4B), three cohort studies involving 1,352 patients were included. These studies provided pre-treatment baseline NLR, with no significant heterogeneity (I^2^ = 20%, *P* = 0.29). The findings indicated that AF patients with stroke exhibited significantly higher NLR values compared to those without stroke (SMD = 0.77, 95% CI = 0.63–0.91, *P* = 0.00001).

#### 3.3.4 NLR and left atrial thrombus

**Figure 5:**
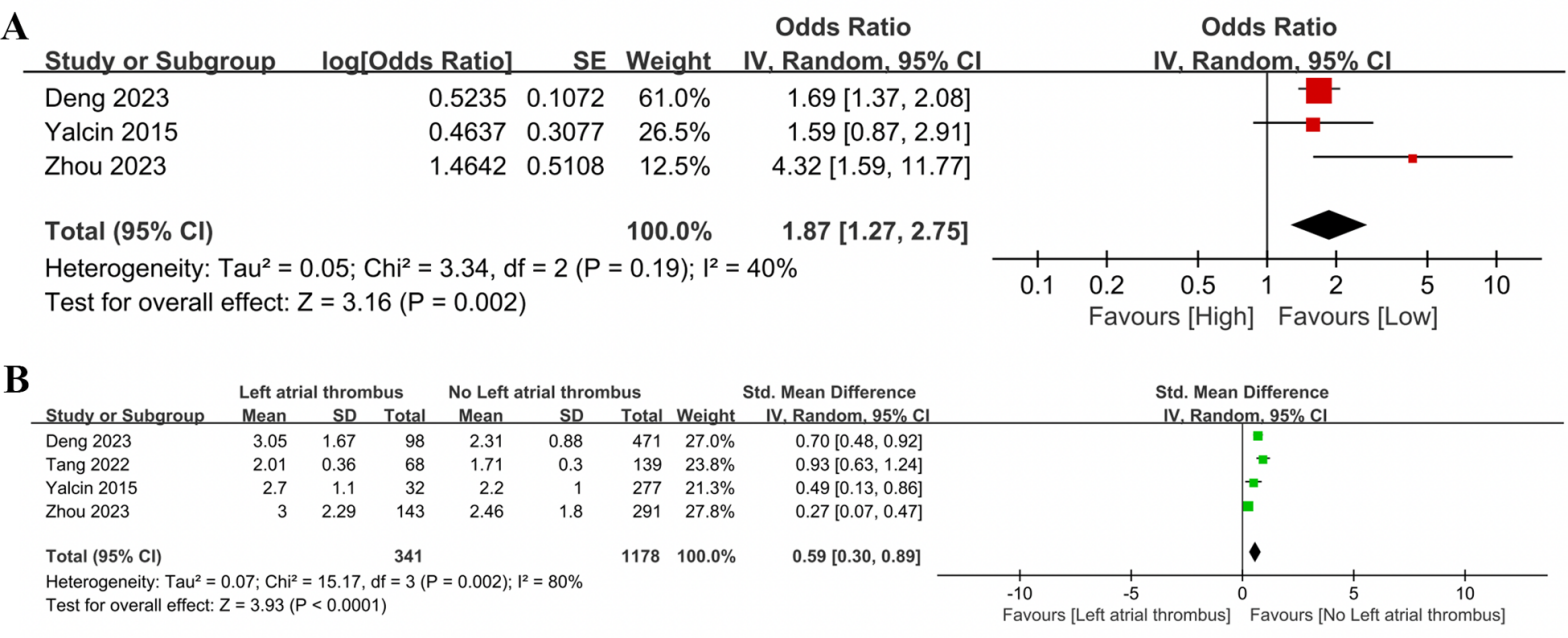
Forest plot of the association between NLR and left atrial thrombus as categorical variable **(A)** and continuous variable **(B).**

Three studies provided the relationship between NLR and left atrial thrombus (categorical variable), all of which were pre-treatment-controlled studies. No significant heterogeneity was detected in the studies (I^2^ = 40%, *P* = 0.19). As shown in Figure 5A, a high NLR was significantly related to left atrial thrombus (OR = 1.87, 95% CI = 1.27–2.75, *P* = 0.002), indicating that a higher NLR was significantly linked to an increased risk of left atrial thrombus in AF patients.

Four controlled studies involving 1,519 patients were included for left atrial thrombus analysis (continuous variable) (Figure 5B). A considerable level of heterogeneity was identified in the studies (I^2^ = 80%, *P* = 0.002). The analysis demonstrated that patients with AF who had left atrial thrombus exhibited significantly higher NLR values compared to those without (SMD = 0.59, 95% CI = 0.30–0.89, *P* = 0.0001).

### 3.4 Sensitivity analyses

**Figure 6:**
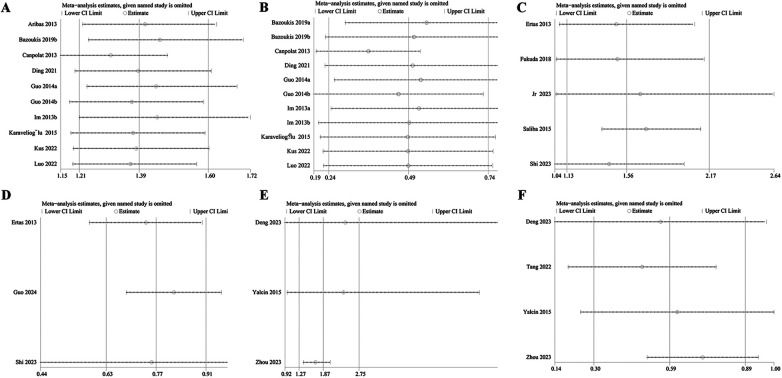
Sensitivity analysis of NLR and recurrence rate as categorical variable **(A)** and continuous variable **(B)**; sensitivity analysis of NLR and stroke as categorical variables **(C)** and continuous variable **(D)** sensitivity analysis of NLR and left atrial thrombus as categorical variable **(E)** and continuous variable **(F).**

The sensitivity analyses indicated that the outcomes of this meta-analysis were robust and reliable, as shown in Figures 6A–F. Additionally, the results for recurrence rate, stroke, and left atrial thrombus were not significantly influenced by any single included study. Due to the limited number of studies on mortality (*n* = 2), no sensitivity analysis was conducted for this outcome.

### 3.5 Publication bias of the studies included

**Figure 7:**
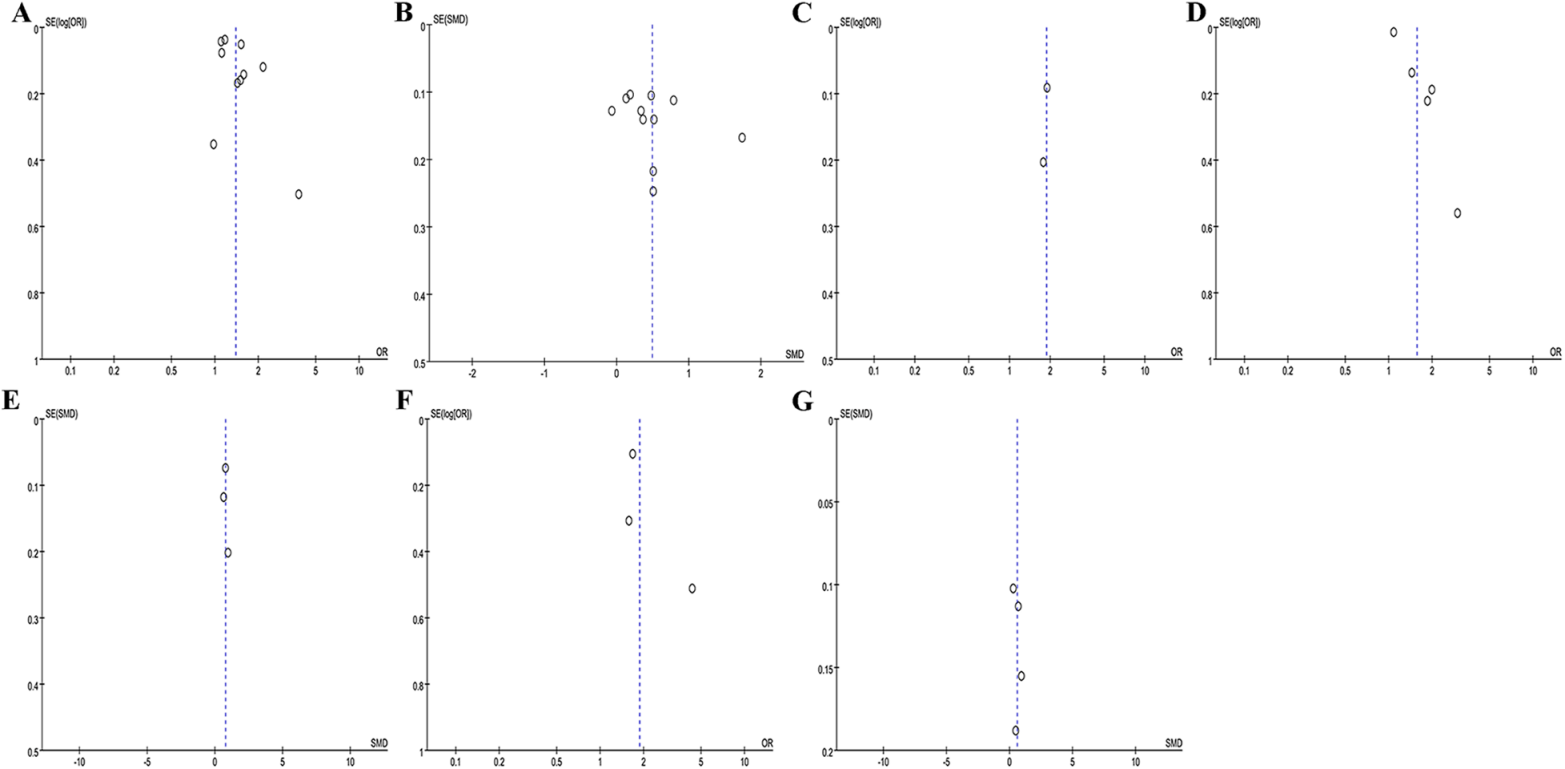
Funnel plot of the association between NLR and recurrence rate as categorical variable **(A)** and continuous variable **(B)**; funnel plot of the association between NLR and mortality as categorical variable **(C)** funnel plot of the association between NLR and stroke as categorical variable **(D)** and continuous variable **(E)**; funnel plot of the association between NLR and left atrial thrombus as categorical variable **(F)** and continuous variable **(G).**

Egger's test and Funnel plots were utilized to assess publication bias. As shown in Figures 7A–G, the funnel plots appeared symmetrical. The Egger's test results were as follows: *P* was 0.129 for recurrence rate (categorical variable), 0.314 for recurrence rate (continuous variable), 0.005 for stroke (categorical variable), 0.904 for stroke (continuous variable); 0.508 for left atrial thrombus (categorical variable), and 0.535 for left atrial thrombus (continuous variable).

## 4 Discussion

Over the last two decades, the occurrence and spread of atrial fibrillation have escalated, and this upward trend is anticipated to persist for the forthcoming three decades, particularly in nations or regions that possess a moderate socio-demographic index. AF has emerged as one of the most significant epidemic and public health challenges we face (35). The pathogenesis of AF is complex and it is caused by multiple factors such as fibrosis, oxidative stress, inflammation, prothrombotic state and genetics, and inflammation is linked to the initiation and perpetuation of AF, resulting in atrial structural abnormalities and electrophysiological changes (1). Recognizing the pivotal role of inflammation in the advancement of cardiovascular diseases, inflammatory biomarkers like the NLR have gained significant interest in recent years (36). Clinical studies have indicated that preoperative NLR is able to predict late recurrence of non-valvular atrial fibrillation (NVAF) (20). However, according to previous studies, the predictive value of NLR for AF patients is not consistent. For instance, in a prospective cohort study conducted by Aribas et al., it was determined that NLR did not serve as a predictor for the recurrence of AF in patients following cardiac surgery (23). Based on the controversy of predicting the prognosis of AF with NLR, we undertook a systematic and exhaustive meta-analysis to clarify that NLR can serve as an effective biomarker for predicting the prognosis of AF patients.

This meta-analysis suggests that high NLR is an important biomarker for poor prognosis in AF and is significantly associated with recurrence rate, mortality, stroke, and left atrial thrombus. Sensitivity analyses and tests for publication bias have substantiated the dependability of our findings. In conclusion, high NLR is an important prognostic biomarker in patients with AF. Undeniably, recent studies on the prognosis of NLR and AF have been published. For example, a meta-analysis by Lekkala et al. found a significant positive correlation between NLR and AF recurrence (14). Lu et al., in a meta-analysis that included 11 studies involving 35,221 patients, found that elevated NLR values were associated with a higher risk of stroke in AF patients (37). Previous meta-analyses have often focused primarily on AF recurrence rates and stroke, with limited reports on mortality and left atrial thrombus. Therefore, our study expanded on this by including all types of AF patients, incorporating 20 studies with a total of 59,256 patients. This analysis not only examined AF recurrence rates and stroke but also included mortality and left atrial thrombus, covering common clinical prognostic indicators. To the best of our knowledge, this is the latest and most comprehensive meta-analysis investigating NLR in the prognosis of AF patients.

During the past few years, various meta-analyses have similarly documented the prognostic function of NLR in various cardiovascular diseases (38, 39). In a meta-analysis by Vakhshoori et al, it was discovered that an elevated NLR level was associated with a notably higher risk of mortality in individuals with heart failure (38). Furthermore, Wang et al. found in their analysis that NLR was a predictive factor for mortality of all cause and cardiovascular events in patients who have undergone coronary angiography or cardiac revascularization procedures (39). In a meta-analysis by Perry et al., which included 12 studies involving 13,262 patients undergoing cardiac surgery, it was found that perioperative NLR is an independent predictor of both short-term and long-term postoperative mortality following cardiac surgery (40). In our analysis, we discovered a notably substantial prognostic influence exerted by NLR on AF, which is consistent with the findings in other cardiovascular diseases.

In order to provide a more detailed analysis, we performed subgroup assessments on AF recurrence and stroke-related factors to explore the relationship between a high NLR and stroke and AF recurrence. Since left atrial thrombus is a contraindication for catheter ablation in patients with AF, patients with left atrial thrombus were excluded by transesophageal echocardiography (TEE) before catheter ablation in our included studies. Due to the limited binary and continuous data on mortality and left atrial thrombus, we did not conduct a subgroup analysis for these outcomes. However, our statistical analysis of categorical variables revealed that a high NLR was significantly associated with an increased risk of mortality and left atrial thrombus in AF patients. The mechanism by which NLR influences the formation of left atrial thrombus in AF patients may be related to neutrophil extracellular traps (NETs) released by neutrophils, which promote thrombosis (41, 42). In addition, lymphocytes have also been shown to regulate thrombosis (43). Subgroup analyses showed significant differences in AF recurrence and stroke before and after treatment, across different follow-up durations, and between different countries/regions. In the treatment method subgroups, NLR remained a significant predictor of outcomes in the ablation subgroup, but it was not a significant predictor in the cardioversion subgroup. On the other hand, based on an NLR cut off of 3, patients with AF who exhibited high NLR experienced a notably higher rate of recurrence in the NLR > 3 subgroups, suggesting that if NLR cut off associated with recurrence rate is observed in clinical practice, NLR > 3 can be used as the observation threshold for AF recurrence. Canpolat et al. conducted a study on the role of preablation NLR in the recurrence of AF in 251 patients who underwent cryoballoon-based atrial fibrillation ablation. They reported that pre-ablation NLR > 3.15 was associated with a 2.5-fold increased risk of AF recurrence (19). In the subgroup of AF stroke, we found that in the NLR ≤ 3 subgroup, NLR was significantly associated with the risk of stroke in AF patients. It suggests that NLR ≤ 3 can be used as the observation threshold for AF stroke if the relationship between NLR levels and stroke in AF patients is observed in the clinical practice. However, due to the limited data in our study, it needs to be further confirmed if it could find a threshold range for AF recurrence and stroke in clinical practice.

NLR, an indicator of the combined inflammatory state of neutrophils and lymphocytes, is considered a reliable biomarker of systemic inflammation. A greater degree of inflammation in AF patients is indicated by higher NLR levels (21). Despite this, the precise mechanism linking the NLR with the prognosis of AF remains to be fully explained, it can be explained from the following aspects: First, neutrophils release a variety of proteolytic enzymes like elastase, myeloperoxidase, and acid phosphatase, resulting in destructive effects on cardiac tissue (44, 45). Second, neutrophils can also promote apoptosis and activate atrial fibroblasts by acting on cardiomyocytes, resulting in increased fibrous tissue (46, 47). Additionally, neutrophils contribute to endothelial dysfunction (ED), which has been shown to play a role in the development and worsening of AF. This may explain the poor prognosis in patients with high NLR values (48). In addition, lymphocytes play various roles in cardiac tissue injury and repair (49). Regulatory T lymphocytes (Tregs), through the increased secretion of inflammatory cytokines, can promote immune system activation and pathological remodeling (50). Cytotoxic lymphocytes have been shown to be associated with epicardial adipocyte death and subsequent AF-related fibrosis (51). In many cardiovascular diseases, a low lymphocyte count is considered an independent indicator of poor prognosis (52). In conclusion, NLR contains information about two leukocyte subtypes and reflects the balance between neutrophil and lymphocyte levels in the body. NLR may be more accurate than a single leukocyte count in predicting the occurrence of diseases and has more clinical predictive value (53).

This analysis has several limitations. First, most of the included studies were retrospective, resulting in inevitable selective bias and confounding factors in this study. Additionally, the majority of these studies were conducted in Asian countries, which might lead to regional selectivity bias. Further studies are needed to validate the prognostic relevance of NLR in non-Asian patients with AF. Thirdly, significant heterogeneity was found in the analysis of prognostic outcome measures among studies. While subgroup analyses in this study helped pinpoint sources of heterogeneity, the inconsistency in NLR cut offs used in the included studies likely contributed to it as well. Therefore, there is a continued need for multicenter, prospective trials to confirm the findings of our meta-analysis. Fourthly, due to significant differences in the treatment methods for the patients included in this study, and unavailable treatment methods from some studies on mortality, stroke, and left atrial thrombosis, and a limited number of studies on mortality and left atrial thrombosis, subgroup analysis by treatment methods is infeasible, which is also one of the limitations of this study. Further research is needed to address such problems.

## 5 Conclusions

To sum up, this meta-analysis revealed that a high NLR was significantly linked to adverse outcomes such as recurrence rate, mortality, stroke, and atrial thrombus in AF patients. This association remained consistent regardless of region and treatment timing, and the results were robust. The study's outcomes imply that, in clinical settings, NLR has potential as a prognostic biomarker for patients with AF, with cost-effectiveness and rich information. However, given the limitations of this study, including the predominance of retrospective studies, high heterogeneity, and potential selectivity bias, future research should focus on larger, multicenter, prospective clinical trials to ascertain the precise link between NLR and the AF patients’ prognosis.

## Data Availability

The original contributions presented in the study are included in the article/Supplementary Material, further inquiries can be directed to the corresponding author.
