# Supplementary material for: Predictive value of neutrophil to lymphocyte ratio for clinical outcome in patients with atrial fibrillation: a systematic review and meta-analysis
Source: Front Cardiovasc Med. 2024 Sep 26;11:1461923. doi: 10.3389/fcvm.2024.1461923 (PMC11464451; doi:10.3389/fcvm.2024.1461923)
Supplement: Supplementary file 1 [file Datasheet1.docx]

Supplementary Material

# Supplementary Tables

## Supplementary Table 1. Search strategy.

| **Pubmed-175** |
| --- |
| (((("Neutrophils"[Mesh]) OR ((((((((((((((Neutrophil) OR (Leukocytes, Polymorphonuclear)) OR (Leukocyte, Polymorphonuclear)) OR (Polymorphonuclear Leukocyte)) OR (Polymorphonuclear Leukocytes)) OR (Polymorphonuclear Neutrophils)) OR (Neutrophil, Polymorphonuclear)) OR (Polymorphonuclear Neutrophil)) OR (LE Cells)) OR (Cell, LE)) OR (LE Cell)) OR (Neutrophil Band Cells)) OR (Band Cell, Neutrophil)) OR (Neutrophil Band Cell))) AND (("Lymphocytes"[Mesh]) OR (((((Lymphocyte) OR (Lymphoid Cells)) OR (Cell, Lymphoid)) OR (Cells, Lymphoid)) OR (Lymphoid Cell)))) AND (ratio)) AND (("Atrial Fibrillation"[Mesh]) OR (((((((((((((((((((((((((Atrial Fibrillations) OR (Fibrillation, Atrial)) OR (Fibrillations, Atrial)) OR (Auricular Fibrillation)) OR (Auricular Fibrillations)) OR (Fibrillation, Auricular)) OR (Fibrillations, Auricular)) OR (Persistent Atrial Fibrillation)) OR (Atrial Fibrillation, Persistent)) OR (Atrial Fibrillations, Persistent)) OR (Fibrillation, Persistent Atrial)) OR (Fibrillations, Persistent Atrial)) OR (Persistent Atrial Fibrillations)) OR (Familial Atrial Fibrillation)) OR (Atrial Fibrillation, Familial)) OR (Atrial Fibrillations, Familial)) OR (Familial Atrial Fibrillations)) OR (Fibrillation, Familial Atrial)) OR (Fibrillations, Familial Atrial)) OR (Paroxysmal Atrial Fibrillation)) OR (Atrial Fibrillation, Paroxysmal)) OR (Atrial Fibrillations, Paroxysmal)) OR (Fibrillation, Paroxysmal Atrial)) OR (Fibrillations, Paroxysmal Atrial)) OR (Paroxysmal Atrial Fibrillations))) |
| **Embase-650** |
| ((Neutrophils or (Neutrophil or Leukocytes, Polymorphonuclear or Leukocyte, Polymorphonuclear or Polymorphonuclear Leukocyte or Polymorphonuclear Leukocytes or Polymorphonuclear Neutrophils or Neutrophil, Polymorphonuclear or Polymorphonuclear Neutrophil or LE Cells or Cell, LE or LE Cell or Neutrophil Band Cells or Band Cell, Neutrophil or Neutrophil Band Cell)) and (Lymphocytes or (Lymphocyte or Lymphoid Cells or Cell, Lymphoid or Cells, Lymphoid or Lymphoid Cell)) and ratio and (Atrial Fibrillation or (Atrial Fibrillations or Fibrillation, Atrial or Fibrillations, Atrial or Auricular Fibrillation or Auricular Fibrillations or Fibrillation, Auricular or Fibrillations, Auricular or Persistent Atrial Fibrillation or Atrial Fibrillation, Persistent or Atrial Fibrillations, Persistent or Fibrillation, Persistent Atrial or Fibrillations, Persistent Atrial or Persistent Atrial Fibrillations or Familial Atrial Fibrillation or Atrial Fibrillation, Familial or Atrial Fibrillations, Familial or Familial Atrial Fibrillations or Fibrillation, Familial Atrial or Fibrillations, Familial Atrial or Paroxysmal Atrial Fibrillation or Atrial Fibrillation, Paroxysmal or Atrial Fibrillations, Paroxysmal or Fibrillation, Paroxysmal Atrial or Fibrillations, Paroxysmal Atrial or Paroxysmal Atrial Fibrillations))).af. |
| **Cochrane-15** |
| ((Neutrophils or (Neutrophil or Leukocytes, Polymorphonuclear or Leukocyte, Polymorphonuclear or Polymorphonuclear Leukocyte or Polymorphonuclear Leukocytes or Polymorphonuclear Neutrophils or Neutrophil, Polymorphonuclear or Polymorphonuclear Neutrophil or LE Cells or Cell, LE or LE Cell or Neutrophil Band Cells or Band Cell, Neutrophil or Neutrophil Band Cell)) and (Lymphocytes or (Lymphocyte or Lymphoid Cells or Cell, Lymphoid or Cells, Lymphoid or Lymphoid Cell)) and ratio and (Atrial Fibrillation or (Atrial Fibrillations or Fibrillation, Atrial or Fibrillations, Atrial or Auricular Fibrillation or Auricular Fibrillations or Fibrillation, Auricular or Fibrillations, Auricular or Persistent Atrial Fibrillation or Atrial Fibrillation, Persistent or Atrial Fibrillations, Persistent or Fibrillation, Persistent Atrial or Fibrillations, Persistent Atrial or Persistent Atrial Fibrillations or Familial Atrial Fibrillation or Atrial Fibrillation, Familial or Atrial Fibrillations, Familial or Familial Atrial Fibrillations or Fibrillation, Familial Atrial or Fibrillations, Familial Atrial or Paroxysmal Atrial Fibrillation or Atrial Fibrillation, Paroxysmal or Atrial Fibrillations, Paroxysmal or Fibrillation, Paroxysmal Atrial or Fibrillations, Paroxysmal Atrial or Paroxysmal Atrial Fibrillations))).af. |
| **Web of Science-234** |
| ((((Neutrophils) OR ((((((((((((((Neutrophil) OR (Leukocytes, Polymorphonuclear)) OR (Leukocyte, Polymorphonuclear)) OR (Polymorphonuclear Leukocyte)) OR (Polymorphonuclear Leukocytes)) OR (Polymorphonuclear Neutrophils)) OR (Neutrophil, Polymorphonuclear)) OR (Polymorphonuclear Neutrophil)) OR (LE Cells)) OR (Cell, LE)) OR (LE Cell)) OR (Neutrophil Band Cells)) OR (Band Cell, Neutrophil)) OR (Neutrophil Band Cell))) AND ((Lymphocytes) OR (((((Lymphocyte) OR (Lymphoid Cells)) OR (Cell, Lymphoid)) OR (Cells, Lymphoid)) OR (Lymphoid Cell)))) AND (ratio)) AND ((Atrial Fibrillation) OR (((((((((((((((((((((((((Atrial Fibrillations) OR (Fibrillation, Atrial)) OR (Fibrillations, Atrial)) OR (Auricular Fibrillation)) OR (Auricular Fibrillations)) OR (Fibrillation, Auricular)) OR (Fibrillations, Auricular)) OR (Persistent Atrial Fibrillation)) OR (Atrial Fibrillation, Persistent)) OR (Atrial Fibrillations, Persistent)) OR (Fibrillation, Persistent Atrial)) OR (Fibrillations, Persistent Atrial)) OR (Persistent Atrial Fibrillations)) OR (Familial Atrial Fibrillation)) OR (Atrial Fibrillation, Familial)) OR (Atrial Fibrillations, Familial)) OR (Familial Atrial Fibrillations)) OR (Fibrillation, Familial Atrial)) OR (Fibrillations, Familial Atrial)) OR (Paroxysmal Atrial Fibrillation)) OR (Atrial Fibrillation, Paroxysmal)) OR (Atrial Fibrillations, Paroxysmal)) OR (Fibrillation, Paroxysmal Atrial)) OR (Fibrillations, Paroxysmal Atrial)) OR (Paroxysmal Atrial Fibrillations))) (Topic) |

## Supplementary Table 2. Quality evaluation of the eligible studies with Newcastle–Ottawa scale.

| **Study** | **Selection** | | | | **Comparability** | | **Outcome** | | |
| --- | --- | --- | --- | --- | --- | --- | --- | --- | --- |
|  | **Representative-ness** | **Selection of**  **non-exposed** | **Ascertainment**  **of exposure** | **Outcome not present at start** | **Comparability on most important factors** | **Comparability on other risk factors** | **Assessment of outcome** | **Long enough follow-up (median≥1 year)** | **Adequacy**  **(completeness) of follow-up** |
| Kus et al.(1) | * | * | * | * | - | - | * | * | * |
| Ding et al.(2) | * | * | * | * | - | * | * | * | * |
| Bazoukis al.(3) | * | * | * | * | - | - | * | * | * |
| Canpolat et al.(4) | * | * | * | * | - | * | * | * | * |
| Guo et al.(5) | * | * | * | * | - | - | * | * | * |
| Luo et al.(6) | * | * | * | * | * | - | * | - | * |
| Im et al.(7) | * | * | * | * | - | - | * | * | * |
| Wu et al.(8) | * | * | * | * | - | - | * | * | * |
| Karaveliog˘lu et al..(9) | * | * | * | * | - | * | * | * | * |
| Aribas et al.(10) | * | * | * | * | - | * | * | - | * |
| Jr et al.(11) | * | * | * | * | - | - | * | * | * |
| Saliba et al.(12) | * | * | * | * | - | - | * | * | * |
| *indicates criterion met; - indicates significant of criterion not met. | | | | | | | | | |

## Supplementary Table 3. Quality evaluation of the eligible studies with Newcastle–Ottawa scale.

| **Study** | **Selection** | | | | **Comparability** | | **Outcome** | | |
| --- | --- | --- | --- | --- | --- | --- | --- | --- | --- |
|  | **Case appropriate** | **Representative-ness** | **Control group selection** | **Determination of control groupt** | **Comparability on most important factors** | **Comparability on other risk factors** | **Ascertainment**  **of exposure** | **Same method of ascertainment for case and controls** | **Non-Response rate** |
| Ertas et al.(1) | * | * | * | * | - | * | * | * | * |
| Guo et al.(2) | * | * | * | * | - | * | * | * | * |
| Shi et al.(3) | * | * | * | * | - | * | * | * | * |
| Deng et al.(4) | * | * | * | * | - | - | * | * | * |
| Fukuda et al.(5) | * | * | * | * | - | - | * | * | * |
| Yalcin et al.(6) | * | * | * | * | - | - | * | * | * |
| Zhou et al.(7) | * | * | * | * | - | - | * | * | * |
| Tang et al.(8) | * | * | * | * | - | * | * | * | * |
| *indicates criterion met; - indicates significant of criterion not met. | | | | | | | | | |
